# Supplementary material for: Near-Infrared Fluorescence Imaging with Indocyanine Green for Robot-Assisted Partial Nephrectomy: A Systematic Review and Meta-Analysis
Source: Cancers (Basel). 2023 Nov 24;15(23):5560. doi: 10.3390/cancers15235560 (PMC10705226; doi:10.3390/cancers15235560)
Supplement: Supplementary file 1 [file cancers-15-05560-s001.zip › cancers-2690824-supplementary.pdf]

Supplementary Material

Near-Infrared Fluorescence Imaging with Indocyanine Green for Robot-Assisted Partial Nephrectomy: A Systematic Review and Meta-Analysis

Carlo Giulioni [1,*], Prashant Motiram Mulawkar [2,3,4], Daniele Castellani [1], Virgilio De Stefano [1], Carlotta Nedbal [1], Nariman Gadzhiev [5], Giacomo Maria Pirola [6], Yu Xi Terence Law [7], Marcelo Langer Wroclawski [8], William Ong Lay Keat [9], Ho Yee Tiong [7], Bhaskar Kumar Somani [10], Andrea Benedetto Galosi [1] and Vineet Gauhar [11]

1 Urology Unit, Azienda Ospedaliero Universitaria delle Marche, Polytechnic University of Marche, 60121 Ancona, Italy
2 Department of Urology, Tirthankar Super Speciality Hospital, Akola 444001, India
3 Government Medical College, Srinagar's Super Speciality Hospital, 3QPX+9GP, Bemina, Srinagar 190010, India
4 Urology Unit, University of Edinburgh, Edinburgh EH8 9YL, UK
5 Department of Urology, Saint-Petersburg State University Hospital, Petersburg 190103, Russia
6 Urology Unit, San Giuseppe Hospital, Multimedica Group, 20123 Milan, Italy
7 Department of Urology, National University Hospital, National University Health System, Singapore 119228, Singapore
8 Hospital Israelita Albert Einstein, São Paulo 05652-900, Brazil
9 Department of Urology, Penang General Hospital, George Town 10990, Malaysia
10 Department of Urology, University Hospitals Southampton, NHS Trust, Southampton SO16 6YD, UK
11 Department of Urology, Ng Teng Fong General Hospital, Singapore 609606, Singapore
* Correspondence: carlo.giulioni9@gmail.com

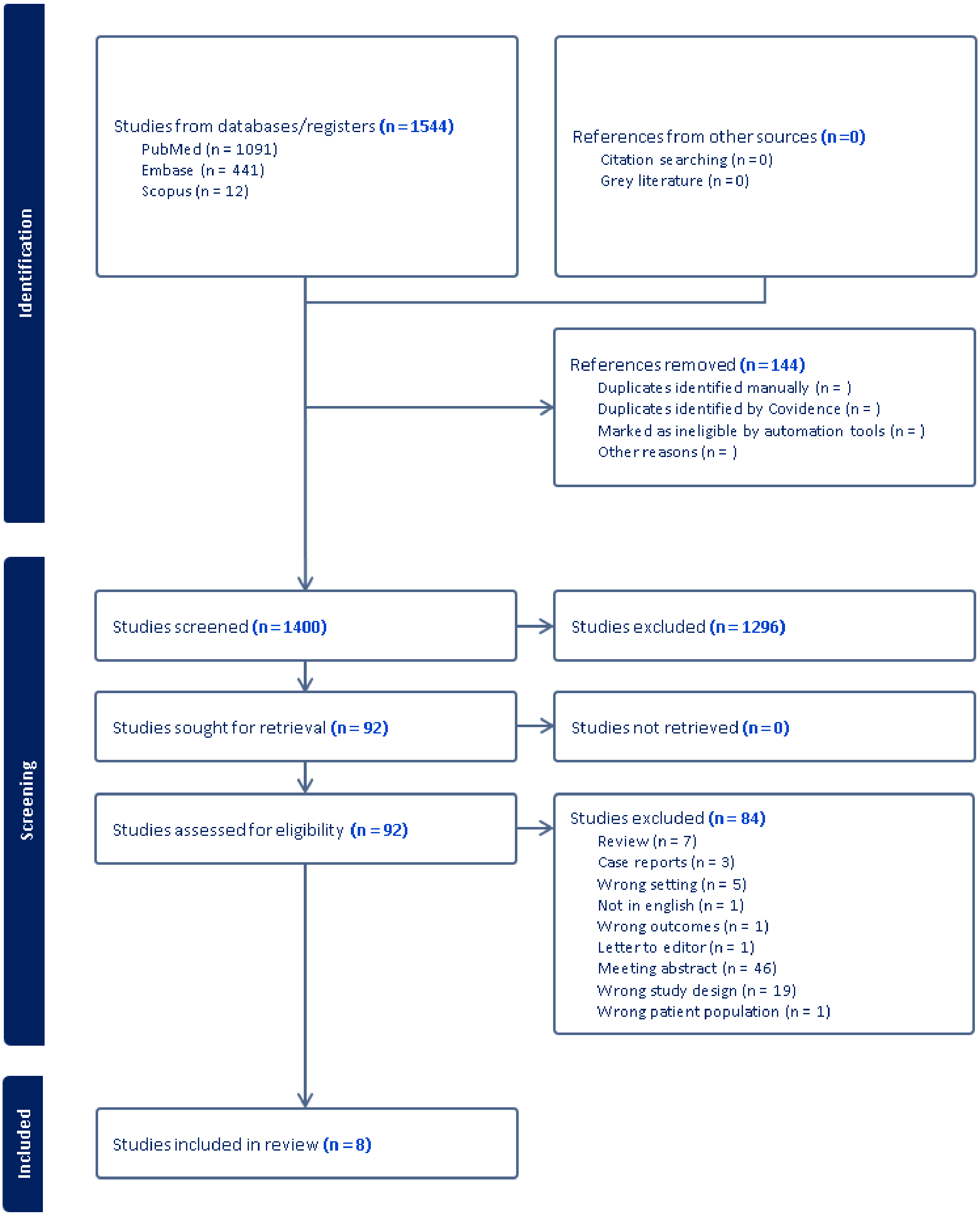

Figure S1. PRISMA 2009 flow diagram.

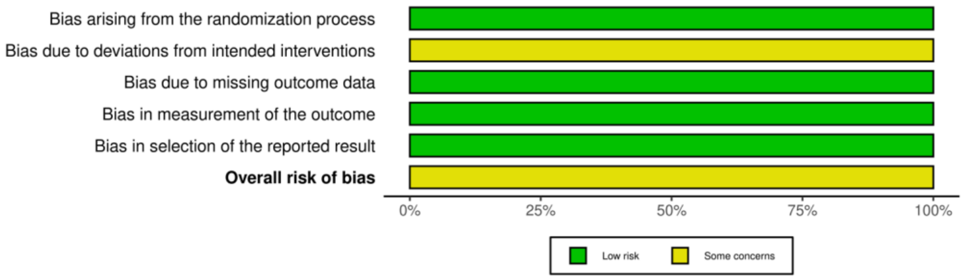

a)

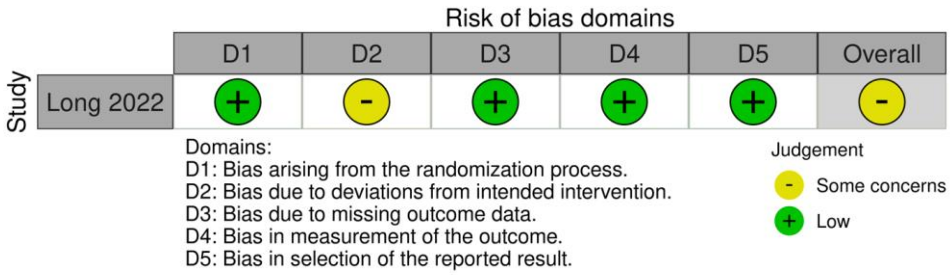

b)

Figure S2. Risk of bias of the included study (Rob2): (**A**) review authors' judgments about each risk of bias item presented as percentages across all included studies; (**B**) review authors' judgments about each risk of bias item for each included study.

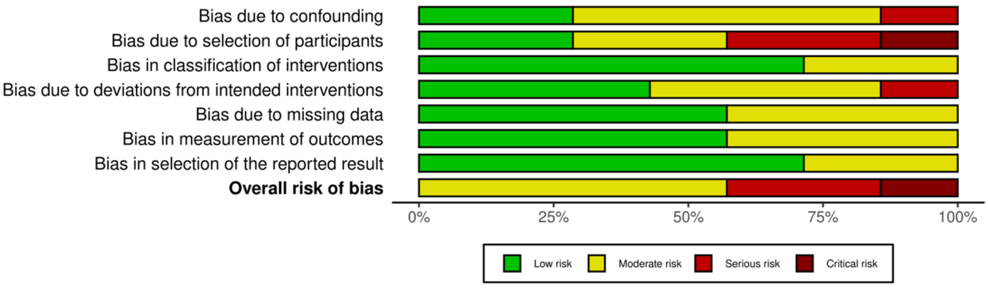

a)

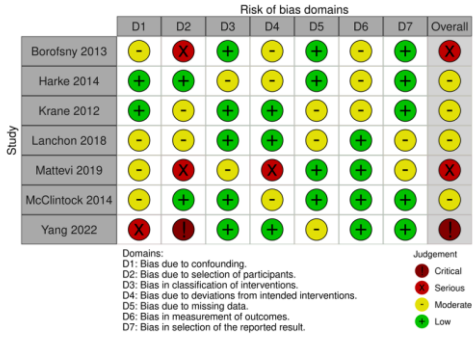

b)

Figure S3. Risk of bias of the included study (ROBINS-I): (**A**) review authors' judgments about each risk of bias item presented as percentages across all included studies; (**B**) review authors' judgments about each risk of bias item for each included study.
